# Editorial: Hardware for artificial intelligence

**DOI:** 10.3389/fnins.2022.979495

**Published:** 2022-08-09

**Authors:** Irem Boybat, Melika Payvand, Oliver Rhodes, Alexander Serb

**Affiliations:** ^1^IBM Research Europe, Rüschlikon, Switzerland; ^2^Institute for Neuroinformatics, University of Zurich, Zurich, Switzerland; ^3^Advanced Processor Technologies Group, Department of Computer Science, The University of Manchester, Manchester, United Kingdom; ^4^Institute for Integrated Micro and Nano Systems, School of Engineering, The University of Edinburgh, Edinburgh, United Kingdom

**Keywords:** artificial intelligence, in memory computing (IMC), noisy hardware, low energy computing, neural networks, neuromorphic computing

The remarkable progress in Artificial Intelligence (AI) has been made possible by a “perfect storm” emerging over the past two decades, bringing together tremendous progress in neuroscience, availability of massive amounts of data, and advents in scalable computer and software systems such as Central Processing Units (CPUs) and Graphical Processing Units (GPUs). However, as AI is increasingly integrated into our daily lives and models grow in scale for solving more complex tasks, its required energy and memory footprint is growing unsustainably. Significant research and development effort is centered around custom hardware solutions targeting low latency, high throughput and better energy savings. Complementary to this, are endeavors toward designing algorithms that are best suited for the underlying hardware. This issue aims to bring together novel research on hardware and algorithms for AI, spanning across a range of applications.

## Hardware accelerators for AI

Memory is the centerpiece of AI hardware research and development, since it occupies the largest area, and is the dominant source of energy consumption in AI systems. The largest energy contribution is related to the data movement between the memory and processing units, known as the von Neumann bottleneck. To minimize this data movement, there is substantial interest in bringing these two units together, known as In-Memory Computing (IMC). Certain computational operations, such as the matrix-vector multiplication that lie at the heart of all neural network operations, can be performed by leveraging Ohm's and Kirchoff's current laws on custom-designed memory arrays, e.g., using Static Random Access Memory (SRAM) and resistive memory. IMC is thus one of the main themes within this issue, featuring also benchmarking efforts for the hardware, including comparative studies across conventional hardware (e.g., CPUs and GPUs) (Steffen et al.) and IMC solutions from a range of hardware/application perspectives (Bagheriye and Kwisthout, Dazzi et al., Kim et al., Lu et al.). Specifically, NeuroSim, a simulator for compute-in-memory hardware accelerators, is presented in Lu et al. The simulator provides design tools for a range of IMC architectures, and linking device, circuit and algorithmic level performance. The simulator is validated against post-layout simulation of an actual 40 nm RRAM-based IMC macro design.

Additional techniques for improving performance of AI algorithms are also presented within the Research Topic. For example, low-power systems exploiting event-driven architectures for in-sensor compute (Stuijt et al.), and always-on systems for edge implementations of AI algorithms (Chundi et al.). Insights on optoelectronic platforms are also provided, leveraging the complementary properties of optics and electronics (Primavera and Shainline).

## Advances in algorithms for AI hardware

While the novel AI hardware solutions proposed above promise significant gains in energy efficiency, it remains a relatively big challenge to map conventional AI algorithms and workflows onto systems with novel substrates and hybrid bit-precision support. Conventional CPU/GPU-based hardware typically makes use of shared memory and message passing to allow implementation of algorithms such as stochastic gradient descent (SGD) for training, and the underlying floating point number representations enable precise and repeatable computation. Systems based on on-chip low-precision memory omit these features by design, thus requiring different hardware-aware algorithms for training and mapping, to realize their full potential.

The TTv2 algorithm is proposed by Gokmen, which builds on previous work to improve the noise tolerance by 100 ×, and reduce the number of device conductance states from 1,000s to 10s (100 ×). The noise tolerance of matrix-vector multiplication is also improved (10 ×), resulting in an algorithm capable of optimizing DNNs close to their ideal accuracy even at extremely noisy hardware settings. Meanwhile neural network training with asymmetric cross-point elements is investigated by Onen et al.. This work demonstrates how device asymmetry can be exploited, rather than updating model parameters in the direction of negative gradients, the total energy of the system incorporating the effects of device asymmetry is minimized, enabling realization of analog deep learning accelerators. Laborieux et al. adapt equilibrium propagation applied to deep conv nets by reducing gradient estimator bias. This allows local learning in systems such as recurrent neural networks, and the ability to unlock the potential of the IMC devices explored in the remainder of the Research Topic. Zhao et al. employ minibatch-SGD to train memristive devices. The research harnesses gradient averaging across the minibatch and stochastic rounding to overcome device non-idealities and vanishing gradients. Memory overheads are kept low through the use of decomposition methods, and the task of reconstructing gradient matrices internally and externally to memristor arrays is explored. The use of a streaming co-processor for training the memristor hardware is investigated, demonstrating the potential to scale up from small proof-of-concept demonstrators to the large-scale AI workflows. The approach to compress gradient information provides an important step toward biologically-plausible batch averaging during long-term learning, and avoids the poor performance experienced when training non-ideal hysteric devices with small batch sizes.

Mapping to hardware Wang et al. explore the mapping of Bayesian Confidence Propagation Neural Networks (BCPNNs) to memristor-based architecture, overcoming the von Neumann bottleneck which limits access to synaptic storage in conventional digital implementations. The implementation harnesses characteristics of the underlying hardware, e.g., using the dopant drift phenomenon of the memristor to simulate the exponential decay of the synaptic state in the BCPNN learning rule. Consistency between the memristor-based solution and software simulations in Matlab is verified, demonstrating the potential of in-circuit analog computation as a route toward real-time brain emulation. Spoon et al. explore the use of Phase Change Memory (PCM) as a substrate for transformer-based deep neural networks (BERT). The work combines noise-aware training to overcome the drift and noise inherent to PCM devices, together with reduced precision (INT6) digital computation in the attention block. By combining these techniques, software-equivalent accuracy is demonstrated, along with a prospective 11.3 × reduction in energy. Overall these works demonstrate that through application of noise-aware training, non-ideal low-precision devices can be trained to produce software-equivalent performance, highlighting the potential of these emerging technologies.

## Outlook

Overall, we observe that regardless of its growth in recent years, the field of AI hardware is still developing at breakneck pace and seems to have a long technological runway yet ahead of it. We note the substantial interest toward building widespread accelerators and general-purpose platforms, while automating the design process of hardware. This is a constantly evolving strand of research targeting mitigation and then ideally exploitation of hardware non-idealities in the pursuit of efficient AI computation. It is with great pleasure that we present a fleeting, yet highly interesting snapshot of the field in this issue and we sincerely hope that you, the reader, finds it instructive, exciting and inspiring for your own future efforts.

## Author contributions

All authors listed have made a substantial, direct, and intellectual contribution to the work and approved it for publication.

## Conflict of interest

Author IB was employed by IBM Research Europe, Rüschlikon, Switzerland. The remaining authors declare that the research was conducted in the absence of any commercial or financial relationships that could be construed as a potential conflict of interest.

## Publisher's note

All claims expressed in this article are solely those of the authors and do not necessarily represent those of their affiliated organizations, or those of the publisher, the editors and the reviewers. Any product that may be evaluated in this article, or claim that may be made by its manufacturer, is not guaranteed or endorsed by the publisher.

